# Effects of baby-friendly practices on breastfeeding duration in China: a case-control study

**DOI:** 10.1186/s13006-020-00334-4

**Published:** 2020-11-03

**Authors:** Yue Zhang, Jinliuxing Yang, Wenhao Li, Nianrong Wang, Ya Ye, Shuangqin Yan, Sumei Wang, Ting Zeng, Zijuan Huang, Fenghua Zhang, Yin Li, Shiyi Yao, Haijun Wang, Scott Rozelle, Tao Xu, Xi Jin

**Affiliations:** 1 National Center for Women and Children’s Health, Chinese Center for Disease Control and Prevention, Beijing, 100081 China; 2 Department of Maternal and Child Health, School of Public Health, Peking University, Beijing, 100191 China; 3 Chongqing Health Center for Women and Children, Chongqing, 400000 China; 4 Ma’anshan Maternal and Child Health Hospital, Ma’anshan, 243011 Anhui China; 5 Liuzhou Maternity and Child Healthcare Hospital, Liuzhou, 545001 Guangxi China; 6 Qingdao Maternal and Children Healthcare Center, Qingdao, 26600 Shandong China; 7 Freeman Spogli Institute for International Studies, Stanford University, Stanford, California 94305 USA

**Keywords:** Exclusive breastfeeding, Baby-friendly hospital initiation, Breastfeeding duration, Infants

## Abstract

**Background:**

The Baby-Friendly Hospital Initiative is generally considered an effective way to promote breastfeeding. Although China has the largest number of baby-friendly hospitals in the world, research on baby-friendly practices in China is limited, and the rate of exclusive breastfeeding (EBF) at 6 months, 20.7%, compared to the 2025 global goal of 50% is low. It is, therefore, important to determine the factors that remain significant barriers to EBF in China. To explore how the key baby-friendly practices affect EBF duration in China, we used a case-control study to compare the effects of baby-friendly-related practices on both EBF and non-breastfeeding (NBF) mothers at 3 months and to investigate the effects of both single and comprehensive baby-friendly practices in promoting EBF duration at 3 months, which is one step toward EBF at 6 months.

**Methods:**

Participants were recruited from four maternal and child health hospitals in western (Chongqing), eastern (Qingdao), southern (Liuzhou), and central China (Maanshan). A total of 421 mothers (245 in the EBF group, 176 in the NBF group) of infants aged 3 months were surveyed through a self-reported questionnaire from April 2018 to March 2019. The experience of baby-friendly practices and breastfeeding during hospitalization were assessed with yes/no questions. Socio-demographic factors that influenced breastfeeding at 3 months were analyzed using bivariate and multivariate logistic regression analyses.

**Results:**

Of mothers in the EBF group, 65.57% reported engaging in at least seven baby-friendly practices compared to 47.72% of mothers in the NBF group. Significantly more mothers in the EBF group engaged in baby-friendly practices than in the NBF group. These practices included “breastfeeding within one hour after birth” (74.29% vs. 59.09%), “breastfeeding on demand” (86.48% vs. 75.00%), and “never use a pacifier” (46.53% vs. 31.25%). After adjusting for confounding variables, we found that the mothers who engaged in fewer than seven baby-friendly practices were about 1.7 times less likely to breastfeed than were those who engaged in seven or more baby-friendly practices (odds ratio [OR] 1.720, 95% confidence interval [CI] 1.106, 2.667). Further, the mothers who did not breastfeed on demand were as likely to not breastfeed up to 3 months (OR 2.263, 95% CI 1.265, 4.049), as were mothers who did not breastfeed during hospitalization (OR 4.379, 95% CI 1.815, 10.563).

**Conclusions:**

These data from hospitals in China suggest that higher compliance with baby-friendly practices may have a positive impact on EBF at 3 months, particularly in terms of promoting the implementation of breastfeeding on demand and breastfeeding during hospitalization in China.

## Background

The benefits of breastfeeding to both maternal and child health are commonly recognized [1]. Thus, one goal of the Global Nutrition Monitoring Framework was to achieve a global exclusive breastfeeding (EBF) rate of 50% among infants less than 6 months of age by 2025 [2]. The Baby-Friendly Hospital Initiative (BFHI) is generally considered as an effective way to promote breastfeeding and the most effective intervention for health systems to improve the breastfeeding rate [3]. The BFHI is a global action launched by the World Health Organization and the United Nations Children’s Fund in 1991 that requires all facilities that apply for the status of “Baby-Friendly Hospital” to provide maternity care to follow the “Ten Steps for the Successful Promotion of Breastfeeding” (hereinafter, “Ten Steps”) and to apply the principles and purposes of the International Code of Marketing Breast-Milk Substitutes (hereinafter, “The Code”) [4, 5]. The Ten Steps put forward 10 practices to support and promote breastfeeding, and The Code prohibits the promotion and marketing of infant formula to pregnant women as well as requiring the fair payment of infant formula by medical institutions according to (but not beyond) market prices.

Many studies suggest that the BFHI and the practices outlined by the Ten Steps have positive impacts in terms of improving national and local breastfeeding rates [6–9] and promoting breastfeeding initiation and duration [10–13]. An intervention study [14] showed that the EBF rate at 3 months in baby-friendly hospitals was significantly higher than that in a control group of hospitals (43.3% vs. 6.4%). Duyan et al. (2007) [15] found that implementation of the BFHI (from November 2002 to February 2003) increased EBF at 6 months 1.3-fold compared to before implementation of the BFHI (November 2001 to February 2002) in Gazi University Hospital. Braun et al. (2003) [12] found that the hazard ratio was 1.66 (95% confidence interval [CI] 1.40, 1.98) for not breastfeeding exclusively at 1 month and 1.55 (95% CI 1.16, 2.07) for discontinuation of any breastfeeding at 4 months among children born before the BFHI in the 1994 cohort compared with children born after the BFHI in the 1999 cohort*.* Research on baby-friendly practices in China is quite limited and focuses mainly on the effect of a single baby-friendly practice [16, 17]. Very few studies assess the comprehensive effect of baby-friendly practices in China. One study in Hong Kong, China [18], which included only six of the Ten Steps (as other practices were considered difficult to measure), found that mothers who experienced one or fewer baby-friendly practices were nearly three times more likely to discontinue breastfeeding than were those who experienced six baby-friendly practices (odds ratio [OR] 3.13; 95% CI 1.41, 6.95). Another study in a baby-friendly hospital (which received approval in 1993) in Shenzhen, China [19], found that any breastfeeding rate at 4 months increased from 24% in 1992 to 57.79% in 1994 and to 65.34% in 1997, and that the EBF rate at 4 months had increased from 36.43% in 1994 to 56.04% in 1997. Nevertheless, the extent to which baby-friendly practices were implemented was not made clear. Research also shows that ethnic and socioeconomic factors can influence the effectiveness of baby-friendly practices on breastfeeding [20]. Because research in China on such practices is limited, studies on the effects of baby-friendly practices on breastfeeding in China are needed, especially in terms of their relationship to the Ten Steps.

Since the 1990s, China has been vigorously implementing the BFHI and has 7036 approved baby-friendly hospitals [21]. According to the BFHI reassessment data, as of 2014, infants born in baby-friendly hospitals account for 69% of the newborn population. A recent survey, however, reports that the proportion of EBF at 6 months is only 20.7% [22], which is much lower than the overall global target (EBF rate at 6 months of 50%) by 2025 [2]. Considering the disparity between the high number of baby-friendly hospitals but low rate of EBF duration in China, it is necessary to determine the factors that remain significant barriers to EBF in China. Exclusive breastfeeding at 3 months is one step toward EBF at 6 months and is not influenced by China’s policy of maternity leave, which allows 98 days. As such, it is more straightforward to study the relationship between baby-friendly practices and breastfeeding duration at 3 months, even though it falls short of understanding factors related to the global goal of EBF at 6 months. Thus, this study will focus on exploring how certain baby-friendly practices in China affect EBF at 3 months.

Some baby-friendly practices have been commonly used in both baby-friendly hospitals and “not-as-baby-friendly” hospitals (i.e., those that have not applied for certification as baby-friendly hospitals). Data from 2012 show that only 6.2% of live births occurred in baby-friendly hospitals in the United States [23]. Nevertheless, more than half of the pregnant women in Maine had engaged in six or seven baby-friendly practices, regardless of whether they delivered in a baby-friendly hospital [24]. A similar situation exists in China. A number of policies for midwifery institutions include the requirement of baby-friendly practices. Moreover, China has been implementing the BFHI for a long time (approximately 30 years), and a large number (7036) of baby-friendly hospitals exist. This makes it difficult to study the baby-friendly practices by comparing baby-friendly hospitals and not-as-baby-friendly hospitals or by comparing the situation before and after the BFHI. In addition, it is difficult for hospitals to adhere completely to the Ten Steps [25]. Therefore, this study will investigate specific baby-friendly practices and how, individually and in combination, they affect EBF duration in China by comparing the engagement of breastfeeding mothers in EBF versus non-breastfeeding (NBF) mothers at 3 months.

The purpose of this study is to compare the differences in engagement to baby-friendly practices between EBF mothers and NBF mothers of infants at 3 months and to specifically evaluate the effectiveness of single or combined baby-friendly practices in promoting the duration of EBF. We hypothesized that baby-friendly practices have a significant impact on EBF at 3 months and that the number of baby-friendly practices engaged in by EBF mothers is significantly higher than that by NBF mothers. Data of this study were extracted from a cohort study of mother-infant interaction conducted by the same study team. The overall cohort study named “the influence of breastfeeding on the establishment of maternal and infant attachment relationships”. It was designed to observe mother-infant interactions during feeding with infants at 3 months and evaluate the influence on the infant’s development at 6 months and mother-infant attachment at 12 months. This case-control study only uses data from the first wave, when the child was 3 months old.

## Methods

### Participants

This research is a case-control study to investigate whether there is an association between baby-friendly practices and NBF at 3 months. The study was conducted in four maternal and child health hospitals in the cities of western (Chongqing), eastern (Qingdao), southern (Liuzhou), and central China (Maanshan). These four hospitals are the largest centers for child health checkups in local areas. The number of children’s checkups in these hospitals in 2018 ranged from 55,000 to 1.3 million. Mothers of infants aged 3 months were recruited for this study when infants were brought in for their health checkups in these hospitals. The staff of the child health clinics introduced this study to the mothers and determined whether they met the inclusion criteria, as described below.

The study participant selection criteria were as follows: (1) resident of the region; (2) experienced full-term delivery (≥ 37 weeks of gestation); (3) single birth; (4) current age of infant was at least 3 months but less than 4 months; and (5) EBF or NBF at 3 months. Participants were excluded from the study if: (1) the infant had a high risk factor, such as preterm delivery, low birthweight, or was a twin; (2) the mother or infant suffered from a serious disease (e.g., chronic disease of infant, maternal complications during pregnancy). To avoid misclassification, a uniform questionnaire was used to determine inclusion/exclusion status in the hospitals, and all investigators were trained by the study team.

Two questions about feeding patterns were used for grouping. One item concerned the mother’s feeding status in the past 24 h. The investigators judged the status of past 24 h as EBF, infant formula feeding, or mixed feeding. Another item concerned the proportion of breast milk that accounts for total food intake in first 3 months of life, which was determined subjectively by the mother. Based on the mother’s answer, the investigators judged the proportion to be ≥90%, ≤ 10%, or > 10% ~ < 90%.

Based on previous studies, the sample size was estimated with an exposure rate of baby-friendly practices of 0.38, OR of 2.5, and loss rate of 10%, resulting in a sample size of 115 for each group. To satisfy the needs of the original cohort study, we expected each hospital to recruit at least 104 eligible mother-infant pairs, of which 52 pairs were EBF and 52 pairs were NBF. Thus, the sample size in both groups of this study exceeded the calculated minimum sample size.

Finally, a total of 421 three-month-old infants and their mothers were enrolled from April 2018 to March 2019, including 245 in the EBF group and 176 in the NBF group. The smaller sample size of the NBF group was due mainly to the fact that there were relatively few mothers who fit the criteria for the non-breastfeeding group. All samples were included in this study, and the proportion of missing data in each questionnaire was less than 10 %.

### Measurements

EBF in this study was classified as only breastfeeding with no food other than drugs and vitamins’ having been fed to the infant in the past 24 h, with breastfeeding as accounting for more than 90% of the total means of food intake in the first 3 months of life. Considering the difficulties of recruiting NBF mothers in the pre-study, the NBF group was defined as the infants’ almost never receiving human milk, either by direct breastfeeding or expressing/ pumping milk; no breastfeeding in the past 24 h; and not breastfed as accounting for more than 90% of the total intake within the first 3 months. In this study, all infants in the NBF group were fed with infant formula.

The assessed variables of this study were obtained from a self-reported questionnaire for mothers that included general socio-demographic information, history of pregnancy and delivery, the infant’s feeding situation at the time of the survey and during the time that she was in the hospital for the delivery of the baby, and the implementation of baby-friendly practices also during the time that she was in the hospital for delivery of the baby. All information was obtained through multiple-choice or yes/no questions.

Socio-demographic variables included the child’s gender, ethnicity, sibling relationship, delivery mode, parents’ age and educational level, family structure and per capita monthly income, delivery mode, and location of hospital, among others.

For baby-friendly practices, 13 yes/no questions were designed to assess the implementation of the BFHI in China as reported by infant’s mothers. Three questions (question one; question six; and question nine) were reverse coded. Most questions were consistent with the Ten Steps; however, some steps that were difficult to measure directly were determined by asking certain key information of mothers. All questions were designed and modified by the study team based on the suggestions of clinical and research experts of the BFHI. The content of the questions is compared with the Ten Steps in Table 1. Step one concerns hospital policy. It was measured by the question, “Did you receive a gift pack with formula from the staff in the hospital?” which is closely related to The Code and partly reflects the implementation of the policies.

**Table 1:** Comparison of Baby-Friendly practices and the items in questionnaire

Ten Steps to Successful Breastfeeding (WHO,1998)	Items in Questionnaire
Step 1: Have a written breastfeeding policy that is routinely communicated to all health care staff.	Q1:Did you receive a gift pack with formula from the staff in the hospital?
Step 2: Train all health care staff in skills necessary to implement this policy.	Q2:During the first skin-to-skin contact, Q2.1 Contact of the newbirth body’s skin with mothers? Q2.2 Lasts for at least 30 min? Q2.3 Within the first hour after birth?
Step 3: Inform all pregnant women about the benefits and management of breastfeeding.	Q3: Did the medical or nursing staff provide you with breastfeeding information?
Step 4: Help mothers initiate breastfeeding within half a hour of birth.	Q4: Was the baby breastfed within the first hour after birth?
Step 5: Show mothers how to breastfeed and how to maintain lactation even if they should be separated from their infants.	Q5: Did the medical or nursing staff help you learn how to breastfeed?
Step 6: Give newborn infants no food or drink other than breast milk unless medically indicated.	Q6: During your stay in the hospital, was the baby fed with food or drink other than breast milk?
Step 7: Practise rooming-in (allow mothers and infants to remain together) 24 h a day.	Q7: While in the hospital, did the baby and you live in the same room?
Step 8: Encourage breastfeeding on demand.	Q8: Did the medical or nursing staff tell you the baby should be fed whenever he/she wants?
Step 9: Give no artificial teats or pacifiers (also called dummies and soothers) to breastfeeding infants.	Q9: Did the baby use a pacifier during the hospitalization?
Step 10: Foster the establishment of breastfeeding support groups and refer mothers to them on discharge from the hospital or clinic.	Q10: Did the medical or nursing staff give you the phone number or other ways to get breastfeeding support if you need?

For Step two, three questions assessed whether the core points of skin-to-skin contact were accurately carried out. Considering that skin-to-skin should be carried out by medical or nursing staff, these questions were used to expose the training and implementation of baby-friendly practices by staff. If a mother answered yes to all three questions, the situation was categorized as having skin-to-skin contact. In regard to Step three, the BFHI policy in China is “Inform all pregnant and delivering women about the benefits and management of breastfeeding” [26]. Our previous study [27] in 17 provinces of China found that the awareness rate of the benefits of breastfeeding among mothers was 96.5–98.4%. Of delivering women, 78.9% received information about breastfeeding before delivery, and 98.5% received breastfeeding education during hospitalization [28]. Thus, the question, “Did the medical or nursing staff provide you with breastfeeding information?” was used in Step three. Step four concerns advocacy of helping mothers to initiate breastfeeding within a half-hour of birth; however, the current study employed the standard used in China and globally, i.e., within 1 h of birth [26].

In addition, the question, “Did you breastfeed your baby during hospitalization?” was included in the survey, which was hypothesized as an independent factor that may have been influencing breastfeeding during the first 3 months of the baby’s life; this question was not included as part of the Ten Steps. This is abbreviated as “breastfeed during hospitalization” in the rest of this paper.

### Statistical analysis

Analyses were conducted using SPSS 18.0. Descriptive statistical analysis was used to analyze the socio-demographic variables both in the EBF and NBF groups. For bivariate association analysis, baby-friendly practices, socio-demographic variables, delivery information, and family factors in these two groups were compared through a chi-square test, and the means of mother’s age and father’s age were compared by a *t* - test. Multivariate logistic regression analysis was used to evaluate the risk factors of not breastfeeding at 3 months. The feeding patterns at 3 months were taken as dependent variables (EBF = 0, NBF = 1); and 10 baby-friendly practices, number of baby-friendly practices, and breastfeeding during hospitalization were entered into the model one by one as independent variables in addition to socio-demographic factors and delivery and family information. The socio-demographic factors, delivery information, and family factors included child’s gender, ethnicity, delivery mode, mother’s age and education level, father’s age and education level, family structure, per capita monthly income, child’s number in the family, and location of hospital. Collinearity diagnostics were used to assess the relationship between the Ten Steps. In the manuscript, the criterion *p* < 0.05 was used to determine statistical significance.

## Results

### Demographic characteristics

As noted, demographic characteristics of the participants are presented in Table 2. A total of 421 mother-infant pairs (53.68% boys and 46.32% girls) were recruited from four study sites (Site A = 120, Site B = 117, Site C = 119, Site D = 65) in this study, and participants were mainly from the Han population (80.29%). There was no significant difference between the EBF group and the NBF group for the variables of gender, ethnicity, child’s number in family, mother’s educational level, family structure, per capita monthly income (chi-square test), and mother’s and father’s age (*t* - test). In the EBF group, 61.63% of mothers reported natural delivery compared to 47.16% in the NBF group (*χ*^*2*^ = 8.691, *p* = 0.003). The difference in the share of participants in the EBF and NBF groups is related to the small number of NBF in the sample chosen from Site D; the small NBF sample size in this site was due to the fact that there were few mothers who had normal deliveries who were not breastfeeding at 3 months.

**Table 2:** Demographic characteristics of participators in EBF group and NBF group (*n* = 421)

Characteristic	Total Sample n(%) /Mean ± Sd	EBF Group	NBF Group	χ2/ t	*p*
**Child’s gender**				0.487	0.485
Boy	226 (53.68)	128 (52.24)	98 (55.68)		
Girl	195 (46.32)	117 (47.76)	78 (44.32)		
**Child’s ethnicity**				0.006	0.940
Han	338 (80.29)	197 (80.41)	141 (80.11)		
Other national	83 (19.71)	48 (19.59)	35 (19.89)		
**Delivery mode**				8.691	0.003
Natural delivery	234 (55.58)	151 (61.63)	83 (47.16)		
Caesarean section	187 (44.42)	94 (38.37)	93 (52.84)		
**Mother’s education level**				6.280	0.179
Completed junior high school or less	53 (12.59)	25 (10.20)	28 (15.91)		
Completed senior high school or technical certificate	75 (17.81)	40 (16.33)	35 (19.89)		
College degree	114 (27.08)	68 (27.76)	46 (26.14)		
University degree	156 (37.05)	95 (38.78)	61 (34.66)		
Graduate degree	23 (5.46)	17 (6.94)	6 (3.41)		
**Mother’s age**	30.00 ± 4.50	30.00 ± 4.04	30.00 ± 5.06	−1.156	0.248
**Father’s education level (n=420)**				10.293	0.036
Completed junior high school or less	53 (12.62)	31 (12.65)	22 (12.57)		
Completed senior high school or technical certificate	76 (18.10)	34 (13.88)	42 (24.00)		
College degree	112 (26.67)	70 (28.57)	42 (24.00)		
University degree	153 (36.43)	90 (36.73)	63 (36.00)		
Graduate degree	26 (6.19)	20 (8.16)	6 (3.43)		
**Father’s age**	31.00 ± 5.63	31.00 ± 6.09	31.00 ± 5.28	−0.322	0.747
**Family structure**				1.310	0.520
Nuclear family	159 (37.77)	98 (40.00)	61 (34.66)		
Extended family	258 (61.28)	145 (59.18)	113 (64.20)		
Single-parent or reconstituted family	4 (0.95)	2 (0.82)	2 (1.14)		
**Per capita monthly income (USD, n=420)**				2.511	0.643
≤ 435	50 (11.90)	27 (11.02)	23 (13.14)		
435- < 725	101 (24.05)	54 (22.04)	47 (26.86)		
725- < 1000	92 (21.90)	54 (22.04)	38 (21.71)		
1000- < 1445	69 (16.43)	42 (17.14)	27 (15.43)		
≥ 1445	108 (25.71)	68 (27.76)	40 (22.86)		
**Children number in family (n=419)**				0.087	0.769
Multiple children	151 (36.04)	89 (36.63)	62 (35.23)		
One-child	268 (63.96)	154 (63.37)	114 (64.77)		
**Location of hospital**				24.41	< 0.001
Site A	120 (28.50)	62 (25.31)	58 (32.95)		
Site B	117 (27.79)	69 (28.16)	48 (27.27)		
Site C	119 (28.27)	59 (24.08)	60 (34.09)		
Site D	65 (15.44)	55 (22.45)	10 (5.68)		

*EBF* exclusive breastfeeding, *NBF* non-breastfeeding

### Bivariate associations of baby-friendly practices in two feeding groups

As shown in Table 3, 58.10% mothers reported engaging in at least seven baby-friendly practices. In the EBF group, 65.57% of mothers reported engaging in at least seven baby-friendly practices, compared to 47.42% of mothers in the NBF group, with the differences between the two groups as statistically significant (OR 2.086, 95% CI 1.403, 3.101).

**Table 3:** The comparison of Baby-Friendly practices between EBF group and NBF group (*n*, %)

Baby-Friendly practices	Total (*n* = 421)	NBF Group (*n* = 176)	EBF Group (*n* = 245)	*X2*	*p*	*OR (95% CI)*
**Numbers of Baby-Friendly practices**						
< 7 practices	176 (41.90)	92 (52.27)	84 (34.43)	28.49	<0.001	2.086 (1.403, 3.101)
≥ 7 practices	244 (58.10)	84 (47.72)	160 (65.57)			
**Specific practices**						
Q 1: recommending formula sample						
No	409 (97.15)	172 (97.73)	237 (96.73)	0.364	0.546	1.451 (0.430, 4.898)
Yes	12 (2.85)	4 (2.27)	8 (3.27)			
Q2: standard skin-to-skin contact						
No	307 (72.92)	130 (73.86)	177 (72.24)	0.136	0.712	0.921 (0.595, 1.426)
Yes	114 (27.08)	46 (26.14)	68 (27.76)			
Q3: providing breastfeeding information						
No	12 (2.85)	7 (3.98)	5 (2.04)	1.387	0.239	0.503 (0.157, 1.612)
Yes	409 (97.15)	169 (96.02)	240 (97.96)			
Q4: breastfeeding within 1 h after birth						
No	135 (32.07)	72 (40.91)	63 (25.71)	10.856	0.001	0.500 (0.330, 0.757)
Yes	286 (67.93)	104 (59.09)	182 (74.29)			
Q5: helping to learn how to breastfeeding						
No	27 (6.41)	16 (9.09)	11 (4.49)	3.613	0.057	0.470 (0.213, 1.039)
Yes	394 (93.59)	160 (90.91)	234 (95.51)			
Q6: feed other food or water						
No	167 (39.67)	61 (34.66)	106 (43.27)	3.170	0.075	0.696 (0.466, 1.038)
Yes	254 (60.33)	115 (65.34)	139 (56.73)			
Q7: rooming-in						
No	24 (5.70)	14 (7.95)	10 (4.08)	2.858	0.091	0.492 (0.213, 1.136)
Yes	397 (94.30)	162 (92.05)	235 (95.92)			
Q8: breastfeeding on demand (n=420)						
No	77 (18.33)	44 (25.00)	33 (13.52)	8.993	0.003	0.469 (0.284, 0.774)
Yes	343 (81.67)	132 (75.00)	211 (86.48)			
Q9: use a pacifier						
No	169 (40.14)	55 (31.25)	114 (46.53)	9.953	0.002	0.522 (0.348, 0.784)
Yes	252 (59.86)	121 (68.75)	131 (53.47)			
Q10: breastfeeding support						
No	231 (54.87)	97 (55.11)	134 (54.69)	0.007	0.932	0.983 (0.666, 1.451)
Yes	190 (45.13)	79 (44.89)	111 (45.31)			
Breastfeed during hospitalization						
No	50 (11.88)	37 (21.02)	13 (5.31)	24.173	< 0.001	4.750 (2.441, 9.246)
Yes	371 (88.12)	139 (78.98)	232 (94.69)			

*NBF* non-breastfeeding, *EBF* exclusive breastfeeding

For specific practices, the differences in the proportions of “breastfeeding within one hour after birth” (EBF group: 74.29% vs. NBF group: 59.09%), “breastfeeding on demand” (EBF: 86.48% vs. NBF: 75.00%) and “never use a pacifier” (EBF: 46.53% vs. NBF: 31.25%) were statistically significant between the two groups (*p* < 0.05). The proportions of “rooming-in,” “helping to learn how to breastfeed,” and “not feeding other food or water” in the EBF group were higher than those in the NBF group but did not reach statistical significance (0.05 ≤ *p* < 0.10).

Similar patterns were seen for “breastfeeding during hospitalization” (OR 4.750, 95% CI 2.411, 9.246). The rate of breastfeeding during hospitalization was 94.69% in the EBF group, which was significantly higher than that in the NBF group (78.98%).

### Multivariate logistic regression for baby-friendly practices in two feeding groups

Multivariate logistic regression analysis was used to evaluate the risk factors that influence the EBF duration of 3 months by controlling for the socio-demographic, delivery, and family factors. When the variables of women who engaged in at least seven baby-friendly practices (= 0) vs. those who adopted fewer than seven baby-friendly practices (= 1) was analyzed by logistic regression, the results indicated that mothers who engaged in fewer than seven baby-friendly practices were about 1.7 times less likely to breastfeed than were those who engaged in at least seven baby-friendly practices (OR 1.720, 95% CI 1.106, 2.667).

To assess ten specific baby-friendly practices, collinearity diagnostics was used. The variance inflation factors (VIFs) ranged from 1.059 to 1.488, which is less than ten, indicating that no collinearity was found in the ten baby-friendly practices. After adjusting for confounding variables in the logistic model (Table 4), the results indicated that mothers who did not engage in “breastfeeding on demand” were less likely to breastfeed at 3 months (OR 2.263, 95% CI 1.265, 4.049).

**Table 4:** Model for logistic regression of specific Baby-Friendly practices (*N* = 421)

Independent variables	*β*	SE	Wald	df	*P*	*OR (95% CI)*		
Child’s gender (base: boy)	0.246	0.220	1.255	1	0.263	1.279	(0.831,	1.968)
Child’s ethnicity (base: han)	0.444	0.321	1.912	1	0.167	1.559	(0.831,	2.926)
Delivery mode (base: natural delivery)	−0.533	0.246	4.712	1	0.030	0.587	(0.363,	0.950)
Mother’s age	0.085	0.045	3.550	1	0.060	1.089	(0.997,	1.190)
Mother’s education level (base: completed junior high school or less)	−0.141	0.152	0.857	1	0.355	0.869	(0.645,	1.170)
Father’s age	−0.029	0.035	0.681	1	0.409	0.972	(0.907,	1.041)
Father’s education level (base: completed junior high school or less)	0.004	0.140	0.001	1	0.975	1.004	(0.763,	1.323)
Family structure (base: nuclear family)			1.246	2	0.536			
Extended family	0.403	1.091	0.136	1	0.712	1.496	(0.176,	12.700)
Single-parent / reconstituted family	0.638	1.083	0.347	1	0.556	1.892	(0.226,	15.819)
Per capita monthly income (base: < 435 USD)	−0.051	0.092	0.310	1	0.578	0.950	(0.793,	1.138)
Single-child family (base: no)	−0.430	0.271	2.513	1	0.113	0.650	(0.382,	1.107)
Location of hospital (base: Site A)			17.056	3	0.001			
Site B	−0.418	0.345	1.471	1	0.225	0.658	(0.335,	1.294)
Site C	−0.082	0.348	0.055	1	0.814	0.921	(0.466,	1.821)
Site D	−1.827	0.455	16.118	1	0.000	0.161	(0.066,	0.393)
**BFHI practices**								
Q 1: recommending formula sample (base: no)	−1.079	0.703	2.352	1	0.125	0.340	(0.086,	1.350)
Q2: standard skin-to-skin contact (base: yes)	0.019	0.255	0.006	1	0.939	1.020	(0.618,	1.682)
Q 3: providing breastfeeding information (base: yes)	−0.193	0.790	0.060	1	0.807	0.824	(0.175,	3.879)
Q 4: breastfeeding within 1 h after birth (base: yes)	0.284	0.268	1.118	1	0.290	1.328	(0.785,	2.247)
Q 5: helping to learn how to breastfeeding (base: yes)	0.867	0.551	2.479	1	0.115	2.380	(0.809,	7.006)
Q 6: feed other food or water (base: no)	0.134	0.261	0.262	1	0.608	1.143	(0.685,	1.907)
Q 7: rooming-in (base: yes)	0.285	0.510	0.312	1	0.576	1.330	(0.489,	3.615)
Q 8: breastfeeding on demand (base: yes)	0.817	0.297	7.566	1	0.006	2.263	(1.265,	4.049)
Q 9: use a pacifier (base: no)	0.267	0.259	1.061	1	0.303	1.306	(0.786,	2.170)
Q10: breastfeeding support (base: yes)	−0.367	0.235	2.433	1	0.119	0.693	(0.437,	1.099)
Constant	−0.935	1.791	0.273	1	0.602	0.392		

Method: Enter, Model goodness of fit:-2 Likelihood =498.019, R squared = 0.150. X^2^ = 67.514, *p* < 0.001

Mothers who did not engage in breastfeeding during hospitalization were more than four times as likely not to be breastfeeding at 3 months, compared with those who engaged in breastfeeding during hospitalization (OR 4.379, 95% CI 1.815, 10.563).

## Discussion

This case-control study evaluated the impacts of baby-friendly practices on EBF duration in China. The number of baby-friendly practices implemented by EBF mothers at 3 months was significantly higher than that of NBF mothers. Notably, “breastfeeding during hospitalization” and “breastfeeding on demand” might be the key practices that break down the barriers to EBF duration to 3 months in China. Examining the implementation of baby-friendly practices may help to determine the key factors that affect EBF duration for 3 months in China, which may be the precondition to and an important step for EBF for 6 months. Thus, there is a need to strengthen the key baby-friendly practices in all baby-friendly hospitals as well as to promote the implementation of the BFHI, a practice that may guide the policy-making of China and other Asian countries.

This study further confirmed the effectiveness of baby-friendly practices and demonstrated that the risk of not breastfeeding at 3 months is associated with the number of baby-friendly practices to which women were exposed. This finding is consistent with the literature [29] that found that there was a “dose-response relationship” between the number of baby-friendly practices to which women were exposed and the likelihood of improved breastfeeding outcomes (early breastfeeding initiation, EBF at hospital discharge, any breastfeeding, and EBF duration). As Nickel et al. [30] reported, being exposed to six BFHI steps was related to the longest median duration of any breastfeeding (48.8 weeks), followed by four or five steps (39.8 week) and then two or three steps (36.4 weeks) [30].

The exact mechanisms by which baby-friendly practices extend the breastfeeding duration, however, are still unclear. One study hypothesized that the Ten Steps have the potential to significantly affect maternal breastfeeding decisions, while another hypothesized that the social-ecological model provides a suitable theoretical framework to explain how baby-friendly practices affect breastfeeding outcomes at multiple levels [31]. Among the various factors, medical and nursing staff played the most crucial role in mothers’ breastfeeding decisions [32].

Baby-friendly hospitals are required to implement the Ten Steps, but there are large differences in how well each step is implemented. Studies show that less than half of baby-friendly hospitals have fully implemented the Ten Steps [20, 33], and very few mothers (2.62%) reported experienced all ten baby-friendly practices in this study. Baby-friendly practices were found to be poorly implemented in another study [34], such as placement of newborns on the mother’s abdomen, rooming-in, immediate breastfeeding initiation, and skin-to-skin contact.

The implementation of baby-friendly practices can be affected by a number of factors. In this study, the well-implemented practices (implementation ≥90%) included “providing breastfeeding information,” “helping to learn how to breastfeed,” “rooming-in,” and “recommending formula sample.” Practices such as “breastfeeding within one hour after birth” (67.93%) and “standard skin-to-skin contact” (27.08%) (implemented in the delivery room), however, were not engaged in by many mothers. Implementation of these practices is influenced predominantly by medical and nursing staff, and, as such, the biggest obstacle to implementation could be limited education or allocation in this regard of medical and nursing staff [35]. Apart from medical and nursing staff, the implementation of “breastfeeding on demand” (81.67%), “not fed other food or water” (39.67%), and “not use a pacifier” (40.14%) are largely affected by the knowledge, attitude, and practice of caregivers. One study reported that mother’s adherence to the Ten Steps was positively associated with a higher likelihood of EBF in hospital (vs. partial breastfeeding) [36]. In our study, we found that less than half of the mothers experienced “receiving the phone number for breastfeeding assistance (breastfeeding support)” in both the EBF and NBF groups. Most maternal and child hospitals in China provide this information in the ward or include them with discharge tips, but sometimes the parents were not aware that they had been informed. Thus, it is suggested that the medical and nursing staff should take a variety of approaches to introducing breastfeeding assistance using both written notification and verbal communication.

The results of this study reveal a few significant factors in EBF duration by 3 months that are partly consistent with other studies. A cross-sectional study among infants under 9 months old in Switzerland reported that rooming-in, breastfeeding within 1 h, breastfeeding on demand, and no pacifier used were associated with longer breastfeeding duration [8]. A study that assessed six baby-friendly practices in Australia also found that only breastfeeding on demand was associated with increased breastfeeding duration among first-time mothers [20]. For “breastfeeding during hospitalization,” the findings supported the hypothesis that breastfeeding during hospitalization was independent of baby-friendly practices and had a significant effect on EBF at 3 months. Therefore, it is necessary to emphasize the importance of breastfeeding on demand and breastfeeding in the hospital in multiple ways to promote the EBF duration in China.

There are several limitations to our case-control study which compared the number and specific implemented steps of baby-friendly practices on EBF and NBF of infants at 3 months of age in China. First, data were collected from child health checkups and might represent the situation of the BFHI only in local regions. To mitigate this, data collection on baby-friendly practices was not limited to a single institution; rather, it was conducted at four municipal maternal and child health hospitals in four cities located within four different provinces. Second, the use of self-report information is susceptible to potential bias in terms of selective reporting or recall bias. The original study of the relationship between breastfeeding and mother-infant interaction did not focus on sensitive information related to baby-friendly practices; thus, it may have partly avoided bias in selective reporting of baby-friendly practices. Third, the questions used for Steps one and two do not fully or accurately reflect the content of the Ten Steps and are specific to the Chinese situation. Therefore, it is necessary to be cautious when comparing this study’s findings with those of other studies. Nevertheless, this study measured the implementation of all Ten Steps from the perspective of mothers.

## Conclusions

This study suggests that higher compliance with baby-friendly practices may have a positive impact on breastfeeding duration and, in particular, promote the implementation of breastfeeding on demand and breastfeeding during hospitalization.

## Data Availability

The data sets used and/or analyzed during the current study are available from the corresponding author on reasonable request.

## Abbreviations

BFHI: Baby-Friendly Hospital Initiative
EBF: Exclusive Breastfeeding
NBF: Not Breastfed
OR: Odds Ratios
95%CI: 95% Confidence Intervals
